# Electrospun Fiber Alignment Guides Osteogenesis and Matrix Organization Differentially in Two Different Osteogenic Cell Types

**DOI:** 10.3389/fbioe.2021.672959

**Published:** 2021-10-25

**Authors:** Robin M. Delaine-Smith, Alice Jane Hann, Nicola H. Green, Gwendolen Clair Reilly

**Affiliations:** ^1^Department of Materials Science and Engineering, Kroto Research Institute, University of Sheffield, Sheffield, United Kingdom; ^2^Department of Materials Science and Engineering, INSIGNEO Institute for in silico Medicine, University of Sheffield, Sheffield, United Kingdom

**Keywords:** bone, extra cellular matirx, mesenchymal stem cells, primary cilia, collagen, polycaprolactone, osteoblasts, tissue engineering

## Abstract

Biomimetic replication of the structural anisotropy of musculoskeletal tissues is important to restore proper tissue mechanics and function. Physical cues from the local micro-environment, such as matrix fiber orientation, may influence the differentiation and extracellular matrix (ECM) organization of osteogenic progenitor cells. This study investigates how scaffold fiber orientation affects the behavior of mature and progenitor osteogenic cells, the influence on secreted mineralized-collagenous matrix organization, and the resulting construct mechanical properties. Gelatin-coated electrospun poly(caprolactone) fibrous scaffolds were fabricated with either a low or a high degree of anisotropy and cultured with mature osteoblasts (MLO-A5s) or osteogenic mesenchymal progenitor cells (hES-MPs). For MLO-A5 cells, alkaline phosphatase (ALP) activity was highest, and more calcium-containing matrix was deposited onto aligned scaffolds. In contrast, hES-MPs, osteogenic mesenchymal progenitor cells, exhibited higher ALP activity, collagen, and calcium deposition on randomly orientated fibers compared with aligned counterparts. Deposited matrix was isotropic on random fibrous scaffolds, whereas a greater degree of anisotropy was observed in aligned fibrous constructs, as confirmed by second harmonic generation (SHG) and scanning electron microscope (SEM) imaging. This resulted in anisotropic mechanical properties on aligned constructs. This study indicates that mineralized-matrix deposition by osteoblasts can be controlled by scaffold alignment but that the early stages of osteogenesis may not benefit from culture on orientated scaffolds.

## Introduction

Mature compact (cortical) bone tissue has a well-organized, hierarchical structure, comprised mainly of collagen fibers and hydroxyapatite minerals (Weiner and Wagner, 1998; Summitt and Reisinger, 2003). These collagen fibers resist tensile forces and are preferentially orientated within a bone lamella (layer), each of which has a different predominant orientation from the adjacent layer. In a mature osteon, lamellae form concentric layers around a blood vessel, and a commonly observed arrangement of the collagen is a helical (“twisted plywood”) structure (Giraud-Guille, 1988). Different bone regions display different dominant collagen orientations that seem to reflect the predominant loading environment, for example, in racehorses, more transverse collagen can be observed in regions subject to compressive loading, and more longitudinal collagen can be observed in regions subject to tensile loading (Riggs et al., 1993; Skedros and Hunt, 2004). During fetal bone growth or after a fracture, immature osseous tissue is initially formed of randomly organized coarse collagen fibers and is mechanically weak. This matrix is remodeled and replaced by organized lamellar bone. Despite the evident importance of collagen fiber alignment in enabling bone to resist fracture, the process of recreating the native anisotropy remains challenging.

A major goal in bone tissue engineering is to provide cell-scaffold constructs to act as bone substitutes for the repair of serious bone defects that will not heal naturally. The scaffold should facilitate cell differentiation toward the osteogenic lineage and guide neo-tissue formation. Both cellular and extracellular matrix (ECM) organizations play critical roles in determining the biological and mechanical properties of the native bone tissue. For example, it has been seen that the aligned collagen acts as a guide for the mineralization of the tissue, with the central axis of hydroxyapatite crystals running parallel to the orientation of the collagen (Nakano et al., 2012), and this alignment plays a critical role in the bone matrix quality and final structural integrity of regenerated bone (Nakano et al., 2012). Ultimately, being able to recreate this structural anisotropy of mature bone is an important goal for the restoration of proper tissue function, quality, and to avoid mechanical mismatch.

Osteogenic progenitor cells are a promising cell source for regenerative medicine strategies, and they can be obtained from numerous sources including bone marrow (Pittenger et al., 1999), adipose tissue (Zuk et al., 2001), and embryonic precursors (Karlsson et al., 2009). In the human body, mesenchymal stem cells (MSCs) do not reside alongside osteocytes (bone-maintaining cells) in mature compact bone, but are involved in healing responses and are known to migrate to sites of inflammation where they assist tissue repair and regeneration. MSC differentiation at the wound site is likely to be initiated and directed by cues from the local microenvironment. These complex environments consist of soluble factors, extracellular forces, cell–cell interactions, and cell–ECM interactions (Lacroix et al., 2002; Wong et al., 2004; Morgan et al., 2008). In a healing bone wound, the ECM resembles a more porous and disorganized structure (e.g., woven) compared with the highly organized mature tissue (lamellar) where mature osteoblasts and osteocytes are present. This prompts the question as to whether a disorganized or more organized substrate would be more supportive of MSC differentiation in a tissue engineered construct.

It has been suggested that manipulation of MSC morphology can affect differentiation by using the cell’s ability to spread or create traction on a surface (McBeath et al., 2004). A number of physical properties have been shown to influence MSC differentiation, including substrate rigidity (Engler et al., 2006; Park et al., 2011; Wen et al., 2014), pore surface topology (Viswanathan et al., 2015), nanotopography (Dalby et al., 2007), and surface roughness (Kumar et al., 2012), while osteogenesis in osteoprogenitor cells and MSCs has been induced using topographical cues (Dalby et al., 2007; Oh et al., 2009; Kumar et al., 2011). However, studies conducted on rigid planar surfaces do not replicate the physiological, mechanical, or structural environment that cells experience *in vivo*, and this affects the way a cell attaches and senses its surroundings, which can result in loss of function (Engler et al., 2008). Additionally, micro- and nano-two-dimensional patterns do not reflect the three-dimensional (3D) fibrous nature of musculoskeletal tissue, and translating these technologies into 3D scaffolds presents a more complex issue.

Fibrous scaffolds have been widely used for influencing the behavior of various cell types, such as fibroblasts (Erisken et al., 2013; Delaine-Smith et al., 2014), periodontal ligament cells (Shang et al., 2010), neuronal and primary Schwann cells (Mukhatyar et al., 2011; Daud et al., 2012), and skeletal muscle myoblast cells (Abarzúa-Illanes et al., 2017). Similarly, fibers have been used successfully to aid the differentiation of MSCs into osteoprogenitor cells; it was also shown that the degree of alignment of the fibers may regulate osteoblast orientation, with cells aligning preferentially along the central axis of the fibers (Wang et al., 2011; Eichholz and Hoey, 2018). This alignment is seen to correlate with the direction of secreted collagen deposition and subsequent mineral deposition (Matsugaki et al., 2015). Electrospinning is a simple and tunable technique, capable of producing nano- and micro-fibrous scaffolds from various polymeric solutions that can mimic both randomly organized and highly aligned ECM architectures. It has been shown that the use of electrospun poly(lactic acid; PLLA) nanofibers can direct the orientation of primary osteoblasts and support subsequent fiber mineralization with increasing alignment (Lee et al., 2020).

Polycaprolactone (PCL) is a bioresorbable aliphatic polyester that has been demonstrated to support MSC proliferation and differentiation into an osteoblastic phenotype *in vitro* (Yoshimoto et al., 2003; Li et al., 2007). Aligned electrospun fibrous scaffolds could be useful for mimicking the orientated collagen fibers that are found in mature lamellar bone, while randomly organized electrospun fibers could be representative of a disorganised woven bone.

Previously, we demonstrated that PCL micro-fiber alignment could strongly influence collagen deposition and organization by mature skin fibroblasts, resulting in controllable construct mechanical properties (Delaine-Smith et al., 2014). Therefore, we hypothesized that these same fibers could be used to control mineralized matrix alignment by MSCs induced along the osteogenic lineage, resulting in a more structurally sound and mechanically strong construct using aligned fibers. To investigate whether any of these scaffold-induced effects were dependent on the cell type, we compared the behavior of a murine osteoblast/osteocyte cell line (MLO-A5) with that of MSC-like cells (hES-MPs).

Finally, we sought to elucidate whether there is a relationship between cells’ response to aligned fiber substrates and the cells primary cilia, a microtubule-based organelle continuous with the cell membrane. Studies have also shown that primary cilia regulate osteogenesis (Hoey et al., 2012; Chen et al., 2016) and mineral deposition in MSCs (Delaine-Smith et al., 2014) and are present in mature bone marrow (Coughlin et al., 2016).

## Materials and Methods

### Cell Culture

Two cell types were utilized in this study (Supplementary Figure 1A): the human embryonic stem cell-derived mesenchymal progenitor cell line hES-MP 002.5 (hES-MP; Cellartis, Gothenburg, Sweden) and the late-stage osteoblast/early-stage osteocyte mouse cell line MLO-A5, kindly donated by Professor Lynda Bonewald (University of Missouri, Kansas City, MO, United States) under a Material Transfer Agreement with the University of Texas. MLO-A5s are a rapidly mineralizing cell line with matrix-deposition behavior that is well-characterized within the existing literature (Kato et al., 2001; Dallas et al., 2008; Lu et al., 2018). MLO-A5s were expanded in basal α-media, which consisted of minimum essential alpha medium (α-MEM; Lonza, Verviers, Belgium) containing 10% FCS, 2 mM L-glutamine, and 100 mg/ml P/S; and hES-MPs were expanded in basal α-media containing 2 ng/ml b-FGF and seeded onto gelatin-coated T-75 flasks. All cells were incubated at 37°C in the presence of 5% CO_2_, and fresh media changes were made every 2–3 days. For experiments, MLO-A5 cells were used between passages 25 and 30 and cultured in basal α-media with 50 μg/ml ascorbic acid-2-phosphate (AA) and 5 mM β-glycerophosphate (βGP), while hES-MPs were cultured in the same media without or with 100 nM dexamethasome (Dex) to induce osteogenesis (Supplementary Figures 1B,C) and used between passages 5 and 8. All reagents were obtained from Sigma-Aldrich (Gillingham, United Kingdom) unless otherwise stated.

### Fabrication of Electrospun PCL Micro-Fibers

Polycaprolactone electrospun sheets were fabricated as previously described (Delaine-Smith et al., 2014). Briefly, solutions of poly(ε-caprolactone; PCL; Mn 80 kDa; Sigma-Aldrich, United Kingdom) were prepared at 15 w% in dichloromethane and stirred at room temperature (RT) for 48 h. Electrospinning was performed at RT whereby PCL solution was loaded into 1 ml hypodermic syringes with a 2 mm diameter blunt tip needle connected to a variable high voltage power supply (0–30 kV) and dispensed using a programmable micro-syringe pump. Solutions projected horizontally toward a variable speed steel rotating drum (diameter 6 cm) covered with aluminum foil and deposited as fibers. For all fibrous scaffolds, solution flow rate was set at 4 ml/h, the voltage was set at 11 kV, and the distance between the collector and needle tip was 20 cm. To generate sheets with no preferred angle of fiber orientation (referred to as “random” fibers), the collector speed was set at 200 rpm, while sheets with highly aligned fibers were collected at a speed of 2,000 rpm. At the end of the process, fibrous sheets were placed under vacuum at RT for 24 h and then stored at 4°C in sealed plastic bags for up to 3 months.

The distribution of fiber diameters and orientations has been previously reported (Supplementary Figure 2).

### Cell Culture on Electrospun Fibers

Electrospun scaffolds were cut to the required size and sterilized using 0.1% peracetic acid at RT for 3 h. Samples were rinsed and wetted thoroughly with PBS and then left to soak in 0.1% gelatin solution overnight before seeding the following day. For the analysis of migration, scaffolds (300 μm thick) were cut into circles (Ø = 30 mm), and hES-MPs were seeded in the center using a steel ring (internal Ø = 10 mm) to contain cells. For the analysis of total DNA, alkaline phosphatase (ALP) activity, matrix deposition and visualization, and imaging of primary cilia, scaffolds (300 μm thick) were cut into smaller circles (Ø = 12 mm) to restrict lateral migration, and hES-MPs or MLO-A5s were seeded centrally in 10-mm steel rings. For the mechanical testing of constructs, scaffolds (thickness 100–150 μm) were cut into rectangular strips (30 mm × 10 mm), and hES-MPs were seeded in 10 mm steel rings. Scaffolds were cultured in separate wells, and MLO-A5s were seeded at 150 k/scaffold and hES-MPs at 100 k/scaffold. After 24 h, steel rings were removed, and the constructs were transferred to new wells where supplemented media were added (D1) followed by fresh media changes every 2–3 days.

### Cell Migration

Assessment of hES-MP migration was determined at days 5, 10, and 15 using MTT (Sigma, United Kingdom) [3-(4,5dimethylthiazol-2-yl)-2,5-diphenyltetrazolium bromide] which stains metabolically active cells. Briefly, cells were washed free of media with PBS and incubated with MTT solution for 40 min at 37°C. Images of the resulting purple formazan salt were captured, and the migration distance of the cell layer was determined using ImageJ plotted as the distance from the center of the cell-seeded ring to the edge of the MTT-stained region that was furthest away.

### Total DNA and ALP Activity

Total DNA (per scaffold) and ALP activity were measured at day 12 for MLO-A5s and days 7, 14, and 21 of culture for hES-MPs using a fluorescent Quant-iT™ PicoGreen® dsDNA Reagent Assay Kit (Invitrogen, United Kingdom). Briefly, cells/constructs were washed free of media with PBS and placed into a micro-tube containing a known volume of a carbonate lysis buffer solution, followed by 1 min vortexing and centrifugation at 10,000 rpm for 5 min. Samples were then stored at 4°C for 24 h. Samples were freeze-thawed three times before a known volume of cell lysate was added to the provided Tris-buffered EDTA solution. The Quant-iT™ PicoGreen® reagent was then added, which binds to the double-stranded DNA in the solution, and fluorescence intensity was recorded using an FLx800 microplate fluorescence reader (BioTek, United Kingdom) using 485 nm excitation and 520 nm emission. Total DNA was converted to nanograms of DNA/sample from a standard curve. ALP activity in the cells was assessed using a colorimetric assay; a known quantity of cell lysate was added to a *p*-nitrophenol phosphate substrate (Sigma), and the subsequent conversion to *p*-nitrophenyl was measured by recording the rate of color change from colorless to yellow at 405 nm. ALP activity was calculated as nanomoles of substrate converted per minute using a standard curve and then normalized to total DNA.

### Collagen and Calcium Deposition

Total collagen production (per scaffold) by the seeded cells was quantified at day 12 for MLO-A5s and days 14 and 21 for hES-MPs by staining with 0.1% Picrosirius red (SR) solution (Sigma, United Kingdom) for 18 h at RT on a platform shaker. The remaining SR solution was washed away with deionized water, and the resulting stain was removed with methanol:0.2 M sodium hydroxide (1:1) for 30 min on a platform shaker. The absorbance of the resulting solution was then measured at 490 nm on a 96-well plate reader. Total collagen was also normalized to ng DNA. Calcium deposition by MLO-A5s and hES-MPs was visualized at days 12 and 21–28, respectively, by staining with a 1% alizarin red (AR) solution for 15 min (Sigma). Excess alizarin solution was removed by washing with deionized water five times, and then, deposited calcium was quantified by removing the alizarin stain with 5% v/v perchloric acid for 10 min and reading the absorbance of the resulting solution at 405 nm.

### Confocal Imaging

Images were captured using a Zeiss LSM 510 Meta upright laser-scanning confocal microscope (Carl Zeiss MicroImaging, Germany) equipped with a 40 × 1.3 NA oil immersion objective. Samples were maintained in PBS and viewed through a coverslip with images captured at a range of depths determined by first moving the focal plane toward the surface of the scaffold until no cells were visible (0 μm) and then moving the focal plane down at set increments. At least, five representative images were captured for each sample. All imaging parameters were optimized (e.g., detector gain and scan speed), and conditions were kept constant for all comparisons.

#### Cell Morphology

Fluorescent staining of cellular morphology was visualized at days 3 and 7 in formalin-fixed (10%) cells using 4',6-Diamidino-2-phenylindole dihydrochloride (DAPI; 1 μg/ml) and Phalloidin-Tetramethylrhodamine B isothiocyanate (Phalloidin-TRITC; 1 μg/ml; Sigma, United Kingdom) for cell nucleus and actin cytoskeleton, respectively.

#### Second Harmonic Generation and Collagen Visualization

Collagen organization was visualized in unfixed hES-MP constructs at days 7, 14, and 21 from second harmonic generation (SHG) images obtained using non-invasive two-photon excitation optimized for collagen detection (29:36). This was achieved using the LSM510 Meta upright confocal microscope attached to a tunable Chameleon Ti:sapphire two-photon laser (Coherent, CA, United States). The illumination wavelength (λI) was set at 940 nm, and SHG emissions were collected in the epidirection with a 10 nm bandpass filter centered around 474 nm after filtration through a primary dichroic (HFT KP650). The objective pinhole was set to maximum, and 20 mW laser power was used.

#### Primary Cilia Imaging

Primary cilia, composed of axonemes containing acetylated α-tubulin microtubule doublets, were visualized in formalin-fixed MLO-A5s and hES-MPs after 24 h of serum starvation at days 3 and 7 by immunostaining with a monoclonal anti-acetylated α-tubulin antibody (produced in mice; clone 611B-1, 1 μg/ml; Sigma-Aldrich) for 24 h at 4°C. Cells were washed, and then, biotinylated anti-mouse IgG (H + L; raised in goat; 2 μg/ml) was applied for 1 h at 20°C, followed by fluorescein isothiocyanate (FITC)-conjugated streptavidin (10 μg/ml; Vector, United Kingdom) for 30 min at 20°C. Images were obtained at a depth of 30 μm, and primary cilia that were ≥ 1 μm in length were measured for their angle of orientation relative to the principle scaffold fiber axis.

### Scanning Electron Microscopy

Electrospun scaffolds without cells were cut into 10 mm circles and mounted on a carbon dot stuck to a metal stub. Samples were then sputter coated with a thin layer of gold under vacuum for 3 min. Samples were imaged using an acceleration voltage of 10 kV and a spot size of 3. To visualize cells and matrix on scaffolds, at days 12 or 21–28 for MLO-A5s and hES-MPs, respectively, constructs were washed with PBS and fixed with 2.5% glutaraldehyde for 1 h. After washing, a series of dehydration steps were performed using ethanol (35, 60, 80, 90, and 100%) for 15 min each followed by treatment with 1:1 ethanol: hexamethyldisilazane (HDMS) for 1 h and 100% HDMS for 10 min. Constructs were then imaged as above.

### Mechanical Testing

Tensile testing of wet PCL fibers without cells or wet hES-MP-seeded constructs cultured with Dex was performed in a biodynamic chamber filled with basal α-medium using a BOSE ELF 3200 equipped with a 22.2 N load cell (ElectroForce Systems Group, BOSE, MN, United States). Separate constructs were tested at day 0 (no cells) and days 2, 7, 14, and 21 (with or without cells) as the tensile test resulted in plastic deformation of the construct. Constructs were measured before testing with a digital micrometer. The gauge distance was set at 12 mm, and samples were strained in displacement mode at a rate of 0.1 mm/s to a maximum strain distance of 6 mm (50% strain) with the resulting force from the scaffold/construct recorded. Stress–strain curves were generated, and Young’s modulus of elasticity (E) was calculated from the gradient of the slope representative of the elastic (linear) region, as well as the tensile stress (TS) recorded at 50% strain.

### Statistical Analysis

All experiments were performed independently at least twice with duplicate or triplicate samples; *n* is defined in figure legends. Collagen organization and mineralized matrix visualization using SHG and scanning electron microscope (SEM), respectively, were performed on duplicate samples (3–5 fields of view) of each fiber orientation per experiment with SHG images being obtained from the central region of the scaffold. Cells cultured on random and aligned scaffolds were compared for statistical differences using a two-way unpaired Student’s *t* test. Where comparisons of more than two sample means were made, one-way ANOVA followed by Tukey’s *post hoc* test was performed. Data are displayed as mean ± 95% CIs or SD where specified, and differences between groups are noted as statistically significant when *p* < 0.05.

## Results

### Electrospun PCL Fibers

Polycaprolactone scaffolds showing two distinct fiber orientations were electrospun, non-aligned (random), or highly aligned as used in our previously described work on fibroblasts (Supplementary Figure 2B). Scaffolds had mean (±SD) fiber diameter of 7.97 ± 1.38 and 8.61 ± 1.28 μm for random and aligned, respectively, and fiber distribution followed a similar pattern (Supplementary Figure 2C); however, the degree of fiber alignment was markedly different (Supplementary Figure 2D). Use of the highly volatile solvent dichloromethane also resulted in the formation of nano-sized pores on the fiber surfaces due to rapid solvent evaporation during the electrospinning process. Altering the duration of spinning time allowed easy control of scaffold thickness. Random scaffolds were ~10% more porous and had a greater water contact angle than aligned scaffolds, which differed depending on the fiber direction (Supplementary Figure 3). Young’s modulus, E, was 10- or 100-fold greater for aligned fibers stretched parallel to the strain direction compared with random fibers or aligned fibers stretched perpendicular to the strain direction.

### MLO-A5 Cells

**Figure 1:**
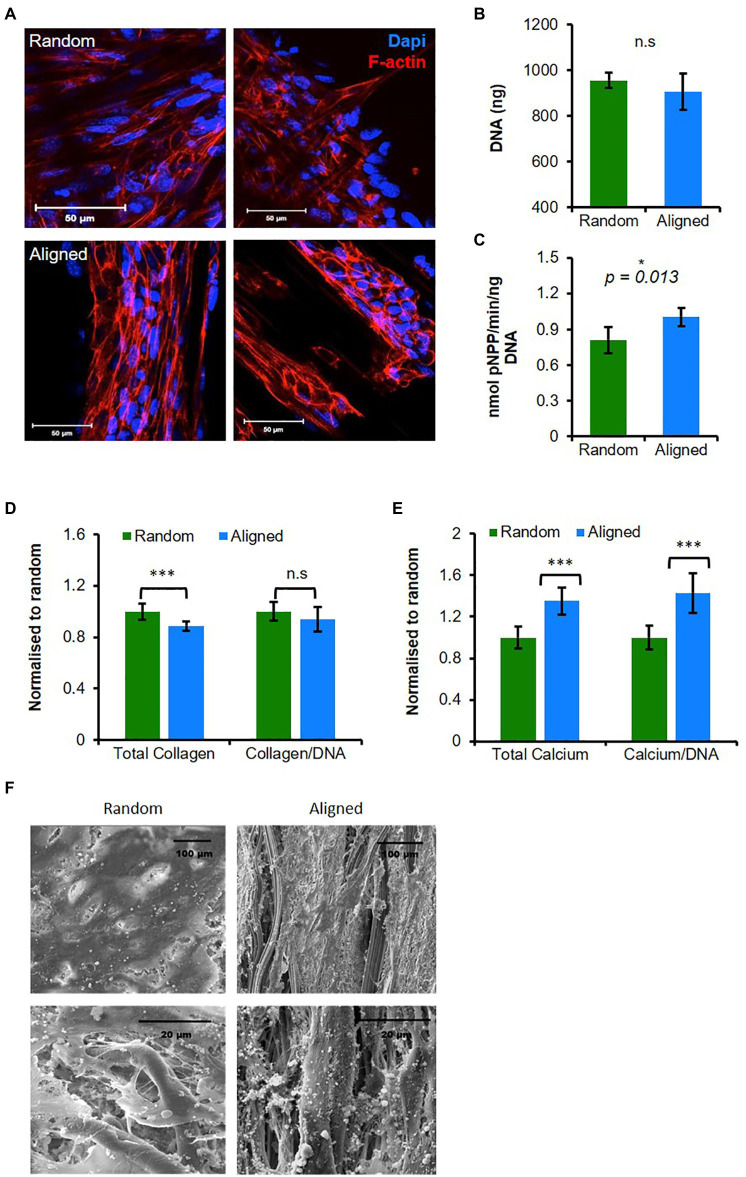
Aligned fibers promote mature osteoblasts (MLO-A5) cell mineralization and organization. MLO-A5-polycaprolactone (PCL) fibrous constructs cultured for **(A)** 3 days and cell nucleus 4',6-Diamidino-2-phenylindole dihydrochloride (DAPI in blue) and cytoskeleton (Phalloidin-TRITC in red) were visualized *via* IF at two depths. Constructs were cultured for 10 days and assayed for **(B)** total DNA, **(C)** Alkaline phosphatase (ALP) activity, **(D,E)** matrix deposition (total and normalized to DNA) measured *via* **(D)** sirius red (SR) staining (collagen) and **(E)** alizarin red (AR) staining (calcium; inserts show stained constructs). **(F)** Representative scanning electron microscope (SEM) images of 10-day-old constructs. Data are mean ± 95% CI for *n* = 9. ^*^p < 0.05, and ^***^p < 0.001 (two-tailed unpaired *t*-test: random vs. aligned).

We first investigated the response of the mature osteoblast cell line MLO-A5s to PCL fiber orientation. Cells seeded onto PCL fibers adopted different morphologies after 3 days depending on scaffold fiber orientation (Figure 1A). On random fibers, cells were more spread and spanned multiple fibers, whereas on aligned scaffolds, cells spread along individual fibers adopting a spindle shape or formed tight cell clusters. Cell number assessed by total DNA was similar for both scaffold orientations at day 12 of culture (Figure 1B).

To assess osteogenic maturation, we measured the ALP activity and ECM production of collagen and calcium. ALP activity normalized to DNA was significantly higher in cells cultured on aligned scaffolds (*p* < 0.05; Figure 1C). Total collagen content assayed by Sirius red (SR) staining was significantly higher on random scaffolds compared with aligned (*p* < 0.05), although the increase was relatively small, when normalized to total DNA, there was no significant difference (Figure 1D). Deposited calcium, an indicator of late-stage osteogenic differentiation, was assessed *via* AR staining identifying significantly higher deposits in aligned scaffolds (*p* < 0.05) showing a >25% increase compared with random scaffolds, and this was also the case when normalized to DNA (Figure 1E).

Scanning electron microscope micrographs collected at day 12 showed that both types of scaffold supported a confluent surface layer of cells, and closer inspection showed that cells had penetrated into the scaffold (Figure 1F). On both scaffolds, thin matrix fibers could be seen, and while there appeared to be no preferential direction of alignment on random scaffolds, on aligned scaffolds matrix appeared to align with the direction of scaffold fibers. Additionally, on aligned scaffolds, there were more visible mineral deposits compared with the random scaffolds.

In summary, PCL fibers controlled MLO-A5 morphology and orientation, while aligned fibers promoted osteogenesis and matrix deposition.

### Human Embryonic Cell-Derived Mesenchymal Progenitor Cells

Next, we wanted to investigate how an osteogenic progenitor cell line, hES-MP, responded to PCL fiber orientation and to assess any similarities with MLO-A5 responses.

#### Cell Migration

**Figure 2:**
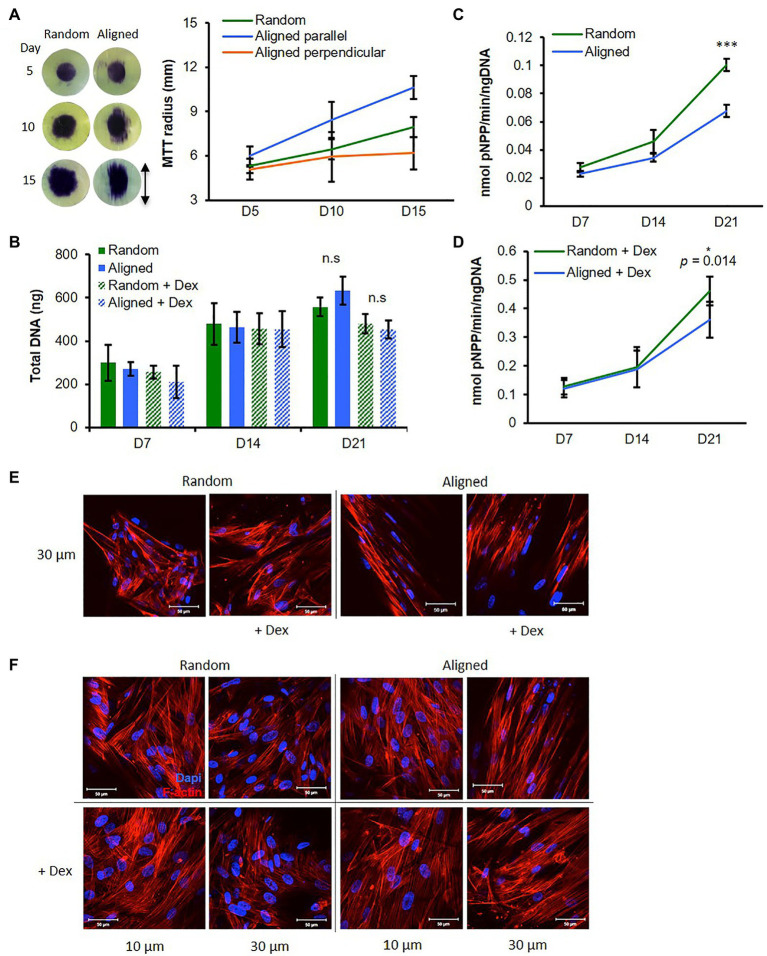
Fiber orientation influences osteogenic mesenchymal progenitor cell (hES-MP) behavior. **(A)** Migration of hES-MPs seeded in center of PCL fibers was assessed at days 5, 10, and 15 using MTT assay (images show MTT-stained scaffolds and arrow indicates aligned fiber orientation). **(B–D)** hES-MP constructs were cultured with or without dexamethasone (+ Dex) for up to 21 days and assayed at days 7, 14, and 21 for **(B)** total DNA, and **(C,D)** ALP activity. hES-MP constructs were imaged for cell nucleus (DAPI in blue) and cytoskeleton (Phalloidin-TRITC in red) at **(E)** day 3 and **(F)** day 7 at two depths. Data are mean ± 95% CI for *n* = 6 (MTT) and *n* = 9 (DNA and ALP). ^*^p < 0.05, and ^***^p < 0.001 (two-tailed unpaired *t*-test: random vs. aligned). Scale bars are 50 μm.

Osteogenic mesenchymal progenitor cells were seeded directly onto the center surface of fibrous scaffolds, and cell coverage was measured over time *via* MTT stain. Cells on random scaffolds showed little migration by day 5 but by days 10 and 15 had spread relatively evenly outward in all directions (Figure 2A). Cells on aligned scaffolds had already begun to spread marginally in the fiber direction by day 5, and by days 10 and 15, cells had spread significantly further parallel to the fiber direction than perpendicular to the fiber direction (Figure 2A). At all time points, cells had spread further parallel to aligned fiber direction than in any other direction on both scaffolds.

#### Deoxyribonucleic Acid, ALP Activity, and Cell Morphology

Osteogenic mesenchymal progenitor cells were cultured on both scaffold orientations either with Dex (+Dex) or without Dex (−Dex). We assessed DNA content (a measure of cell number) across 21 days and saw an increase from days 7 to 14 with similar levels at each time point for all samples (Figure 2B). This suggests that cells in all conditions proliferated from days 7 to 14. At day 21, DNA content was unchanged compared to day 14 for both constructs +Dex, while there were further increases for both constructs −Dex; however, there were no significant differences between fiber configurations within Dex groups.

Alkaline phosphatase activity increased in all constructs from days 7 to 21 (Figures 2C,D) indicating that both scaffold orientations supported osteogenesis. ALP activity was greater for hES-MPs +Dex compared with hES-MPs −Dex at all time points, as expected. For all constructs, the increase in ALP activity between days 14 and 21 was greater than between days 7 and 14, suggesting a greater commitment to differentiation at this later time point. For cells cultured +Dex, ALP activity was significantly higher at day 21 in random fibrous scaffolds compared with aligned fibrous scaffolds (*p* < 0.05). For cells cultured −Dex, ALP activity on random fibers was higher at days 14 and significantly more so at day 21 compared with aligned fibrous scaffolds (*p* < 0.001). Interestingly, hES-MPs cultured on both PCL scaffold configurations produced higher ALP activity than when cultured on 2D tissue culture plastic even without Dex (Supplementary Figures 4A,B).

Similarly to MLO-A5 cells, hES-MPs were well spread on random scaffolds spanning multiple fibers, whereas on aligned scaffolds, cells elongated along individual fibers adopting a narrow spindle shape (Figure 2E). Additionally, hES-MPs cultured +Dex appeared larger on both scaffold orientations compared with −Dex counterparts, a morphological feature typical of osteogenic induction (Figure 2F).

#### Collagen and Calcium Deposition

**Figure 3:**
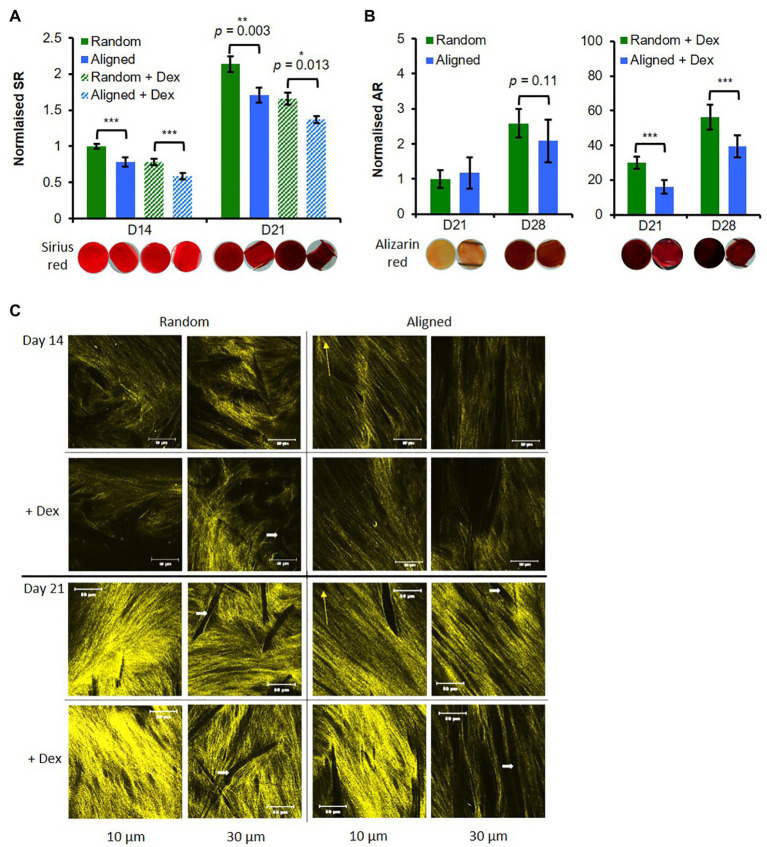
Random fibers promote hES-MP matrix deposition. hES-MP constructs were cultured with or without dexamethasone (+ Dex) for up to 28 days and stained **(A)** at days 14 and 21 with SR for collagen, and **(B)** at days 21 and 28 with AR for calcium deposition; inserts show representative stained constructs for each condition. **(C)** hES-MP constructs were imaged at days 14 and 21 *via* second harmonic generation (SHG; pseudo-yellow) to visualize collagen organization at two depths; yellow arrow indicates PCL fiber orientation and white arrow indicates dark space occupied by PCL fibers. Data are mean ± 95% CI for *n* = 9. ^*^p < 0.05, ^**^p < 0.01, and ^***^p < 0.001 (two-tailed unpaired *t*-test: random vs. aligned or random + Dex vs. aligned + Dex). Scale bars are 50 μm.

Matrix production, a later stage differentiation event, was assessed at days 14 and 21 for collagen and at days 21 and 28 for deposited calcium. Collagen production increased approximately 2-fold for all constructs from days 14 to 21 (Figure 3A). Collagen production was significantly higher on random scaffolds than aligned scaffolds for both Dex conditions at both time points (Figure 3A) and cells -Dex had more collagen than cells +Dex for each respective scaffold type.

All constructs showed increased calcium deposition from days 21 to 28 (Figure 3B). As expected, constructs cultured +Dex for both fiber orientations showed stronger deposited calcium staining at both time points compared with constructs -Dex (>10 fold). For constructs +Dex, calcium deposition was significantly higher (*p* < 0.001) on random scaffolds than aligned scaffolds at both time points. There was a very little observable stain for either construct cultured -Dex at day 21; however, by day 28, there was a noticeable increase in calcium staining on both fiber configurations (Figure 3B) resulting in a 2.5-fold and 2-fold mean increase for random and aligned configurations, respectively. While there was no statistical difference between construct orientation for cells cultured -Dex, random fibers supported more consistent mineral deposition than aligned fibers as evidenced by the smaller SE.

To further explore collagen deposition and organization, SHG signal was collected at days 7, 14, and 21 at two different construct depths (10 and 20 μm; Figure 3C; Supplementary Figure 5). At day 7, random constructs -Dex produced very faint SHG (Supplementary Figure 5C), while all other constructs were absent for SHG signal. SHG signal was detected in all constructs at day 14, and differences in collagen organization were observed. On random PCL fibers, collagen appeared disorganized compared with aligned scaffolds, which appeared to suggest a preferential direction of collagen orientation. Interestingly, at a depth of 10 μm, collagen fibers deposited on aligned scaffolds were not orientated with the scaffold fiber direction but appeared offset at an angle no greater than 45 degrees. SHG intensity was greater on random scaffolds than on aligned scaffolds at a depth of 30 μm. SHG signal intensity increased from days 14 to 21 for all constructs indicating greater collagen deposition over time in agreement with the previous SR staining. At day 21, collagen deposited on random fibers again showed no preferential organizational direction in contrast to collagen on aligned fibers. A similar trend was observed on aligned fibers seen at day 14 whereby collagen appeared organized at an angle offset from the principal direction of scaffold fiber orientation. SHG intensity was higher for random scaffolds compared with aligned scaffolds, especially when cultured +Dex. In contrast to the previous SR staining, there did not appear to be much difference between constructs cultured +Dex and −Dex, suggesting that +Dex may be better organized into thicker bundles.

#### Mineral Organization and Construct Mechanical Properties

**Figure 4:**
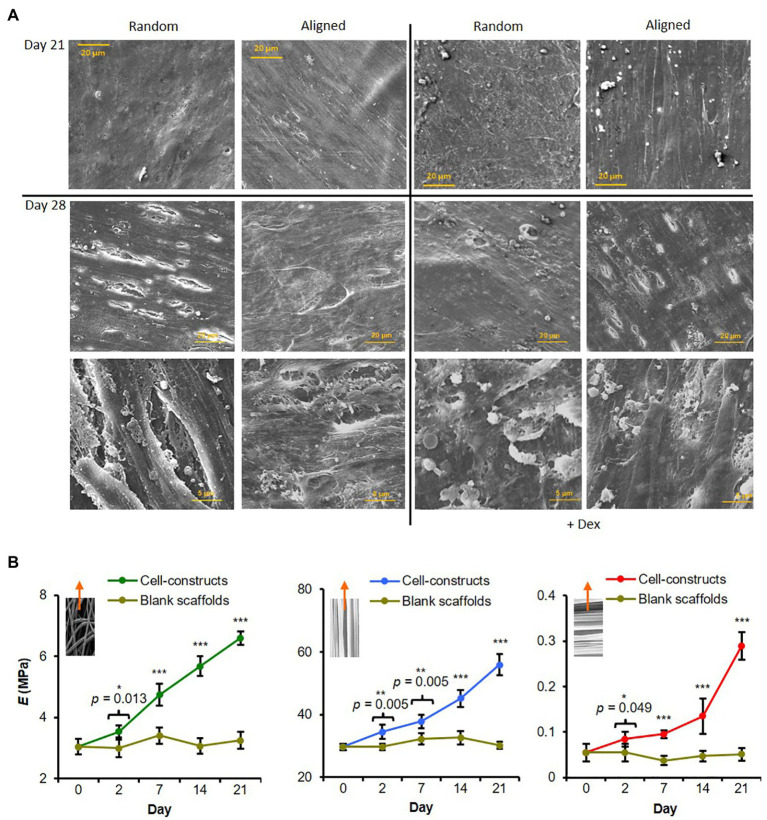
Fiber alignment dictates degree of hES-MP matrix anisotropy and construct modulus. **(A)** hES-MP constructs were cultured with or without dexamethasone (+ Dex) for up to 28 days and imaged at days 21 and 28 *via* SEM. **(B)** PCL scaffolds without cells (blank) and hES-MP constructs were cultured with dexamethasone for up to 21 days, and then, pseudo-static tensile tests were performed on unfixed scaffolds and constructs at days 0, 2, 7, 14, and 21 to calculate the Young’s Modulus (*E*); inserts show direction of tensile tests with respect to PCL fiber orientation; random, aligned parallel, and aligned perpendicular, respectively. Data are mean ± 95% CI for *n* = 5–6. ^*^p < 0.05, ^**^p < 0.01, and ^***^p < 0.001 (two-tail unpaired *t*-test: cell-constructs vs. blank scaffold for each time point).

A SEM was used to visualize surface mineral deposition. All hES-MP constructs were completely covered with cells and matrix by days 21 and 28 (Figure 4A). Cells and matrix on aligned scaffolds appeared to have a higher degree of orientation than on random scaffolds, especially when cultured +Dex. At day 21, constructs cultured -Dex had a relatively smooth appearance, while constructs cultured +Dex had a rough surface with some visible immature mineral particles. On random scaffolds in particular, there appeared to be many particulate bumps residing under or on the top layer of cells and matrix. At day 28, constructs cultured +Dex showed an increase in the occurrence and size of the mineral-like particles. Some small potential mineral particles were also observed on constructs cultured -Dex, agreeing with the faint AR staining observed, indicating that cells were potentially beginning to deposit calcified matrix. At both time points, deposited mineral appeared better organized on aligned scaffolds.

Tensile testing of hES-MP seeded constructs +Dex was performed at days 0, 2, 7, 14, and 21 (Figure 4B) as well as on blank (cell-free) scaffolds in order to monitor any changes in construct mechanics attributed to cells and matrix. Aligned constructs were tested along two different axes, namely, parallel or perpendicular to the principle scaffold fiber direction. At day 2, all constructs had significantly higher elastic modulus, E, compared with blank constructs, which did not change significantly with time. Constructs of all fiber orientations generally showed increasing Young’s modulus across all time points tested over the culture period. Constructs tested with PCL fibers orientated parallel to the strain direction had the highest modulus at day 21 compared and also had the highest absolute increase in modulus across the culture period. In contrast, constructs tested with PCL fibers perpendicular to the strain direction had the lowest modulus at day 21 and the lowest absolute increase across the culture period. Random constructs were >20-fold stiffer than aligned constructs tested perpendicular to the strain but ~8-fold less stiff than aligned constructs tested parallel to the strain. This indicates that cells and deposited matrix reinforce PCL fibers in the direction of principle alignment to form anisotropic constructs.

**Figure 5:**
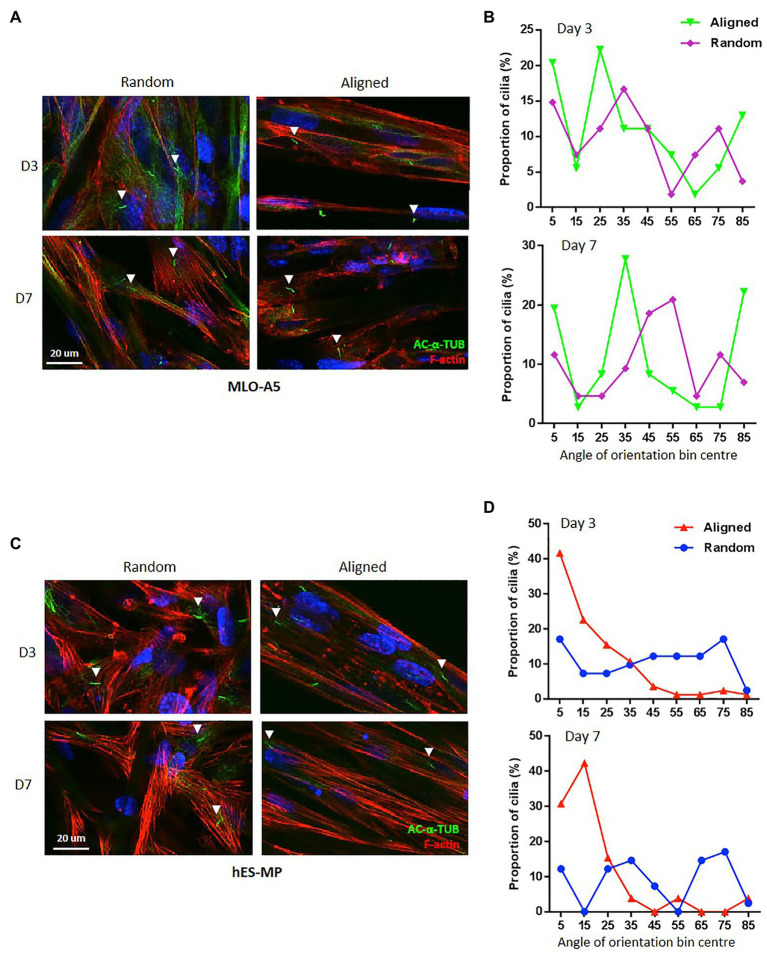
Aligned scaffolds promote alignment of hES-MP primary cilia. **(A,B)** MLO-A5 or **(C,D)** hES-MP constructs were cultured for up to 7 days and imaged at days 3 and 7 *via* IF for cell nucleus (DAPI in blue), cytoskeleton (Phalloidin-TRITC in red), and primary cilia (acetylated-α-tubulin in green). **(B,D)** Primary cilia orientation was measured from IF images relative to a fixed PCL fiber axis of orientation for **(B)** MLO-A5 constructs and **(D)** hES-MP constructs and plotted as proportions in bins 10^0^ wide (primary cilia = *n* ≥ 26 from five images). White arrow heads indicate examples of individual primary cilia.

We identified primary cilia in both cell types cultured on PCL fibers and measured their angle of orientation relative to the principle axis of scaffold fibers (Figures 5A–D). With regard to random fibrous constructs, primary cilia had no preferential direction of orientation for either cell type, and this was true for both days 3 and 7; however, we did observe differences between the two cell types when cultured on aligned fibers. Interestingly, the majority (60–70%) of hES-MP primary cilia were aligned in the same direction and displayed an orientation preference similar to the principal axis of scaffold fibers at both days 3 and 7 (0–20°). This was not the case for MLO-A5s; they displayed multiple orientation preferences relative to the scaffold fiber principle axis, 5° for 85° for both time points as well as 25 and 35° for days 3 and 7, respectively. Therefore, hES-MPs appear to show a high degree of alignment between primary cilia and F-actin fibers on aligned PCL, whereas MLOA5s do not.

## Discussion

The ultimate goal of bone TE strategies is to fabricate a cell-scaffold construct that facilitates progenitor cell differentiation to a specific lineage and guides functional neo-tissue formation. To develop tissue-engineered constructs that are suitable for implantation, both physiological function and tissue mechanical properties must be replicated. To achieve this, scaffold materials should mimic the native ECM, in order to control cell behavior and differentiation and to match the biological function of the *in vivo* tissue.

The main aim of this study was to observe what influence micro-fiber orientation (no specific orientation vs. aligned) had on the behavior and differentiation of the osteogenic progenitor cells and the effect on matrix organization. We hypothesized that aligned fibers being more similar to those within bone lamellae would support mineralized matrix deposition. This study indicates that substrate fiber orientation can influence the deposited matrix organization of both progenitors and mature osteogenic cells. Surprisingly, osteogenic differentiation and mineralization of progenitor cells were better stimulated on random fiber orientations. However, this is not a general phenomenon as under matching conditions aligned fibers appeared to support mineralization by MLO-A5 mature bone cells more so than random fibers.

As expected, aligned fibers caused both cell types to orientate along the principal direction of fiber alignment, as has previously been observed for multiple different cell types (Zhong et al., 2006; Delaine-Smith et al., 2014; Lee et al., 2020). Both fiber orientations supported osteogenic differentiation and matrix deposition by Dex-treated hES-MPs. In addition, Dex-stimulated ALP activity was higher in electrospun scaffolds than in monolayer on tissue culture plastic (Supplementary Figure 2). Interestingly, even in non-Dex containing media, cellular ALP activity was upregulated on scaffolds compared to traditional tissue culture plastic, confirming other research that a 3D environment favors differentiation. However, the random fibrous configuration appeared to favor osteogenesis of hES-MPs compared with aligned fibers, showing significantly increased ALP activity, collagen, and calcium deposition.

In contrast to this, calcium deposition by MLO-A5 cells was significantly higher on aligned fibers. It may be noted that MLO-A5 cells experience spontaneous mineralization in culture, without the presence of β-GP and ascorbic acid (Kato et al., 2001). It is also known that MLOA-5 cells tend to deposit mineral in sheets, and in a study by Dallas et al. (2008) using time lapse imaging of MLO-A5’s mineral deposition compared to primary osteocytes, it could be seen that MLO-A5 cells deposited mineral in association with collagen fibrils, as opposed to discrete nodules as is seen by primary osteoblasts in culture.

In both cases, aligned constructs supported a higher degree of mineralized matrix organization, and this contributed to the 8-fold increase in stiffness for constructs tested parallel to principal scaffold alignment, supporting previous theories that it is mineral organization over quantity that determines overall strength.

There is a large wealth of evidence indicating that cell behavior and differentiation are heavily influenced by cell morphology and spreading (Chen et al., 1997; Bhadriraju et al., 2007). MSCs that spread out over a larger area have been seen to favor osteogenesis compared with those that occupy a small area favoring adipogenesis (McBeath et al., 2004). Cells that spread out over a large area are able to form numerous focal adhesion complexes that geometrically oppose one another and hence generate significant cytoskeletal tension. It has been suggested that MSCs must develop cytoskeletal tension above a threshold value in order to undergo osteogenic differentiation (Rowlands et al., 2008), and also be bound to an osteogenic ligand. In this study, both fiber orientations supported well-spread cells with well-defined actin stress fibers. Well-spread MSCs express common osteoblastic markers associated with calcifying bone proteins, and this is dependent on the cell being able to apply traction to the material. Induced cellular orientation along well-organized fibers has been reported in a number of cell types, and it is believed that various integrins are involved in this fiber-induced cell adhesion. It has also been seen that human adipose-derived stem cells seeded on random fibrous mats possessed short and random focal adhesion plaques, whereas cells on aligned fibers had long focal adhesion plaques orientated in the fiber direction (Fu and Wang, 2012). PCL fibers also contained nano-pores on the fibers (Delaine-Smith, 2013), giving the surface an inherent roughness, and this has also shown to be favorable for osteogenesis compared with smooth surfaces (Woo et al., 2003; Kumar et al., 2011; Patel et al., 2019) and could explain why PCL constructs cultured -Dex displayed some calcium deposition at day 28.

As mature bone consists of alternating layers of parallel-aligned collagen fibers, it would be reasonable to speculate that the use of aligned fibrous scaffolds mimicking the native tissue would better enhance osteogenesis. But this study indicates that random fibers better supported the osteogenic differentiation of hES-MP cells compared with aligned fibers. There are a number of previous studies that have examined the effect of fiber properties on MSC differentiation but results have been conflicting. For example, hMSCs seeded on aligned collagen thread scaffolds had a significantly lower osteocalcin expression at day 14 compared with hMSCs seeded on random threads (Kishore et al., 2012). Lü et al. seeded mouse bMSCs on random macro- and random and aligned nano-fibers with random nanofibers showing the best cellular compatibility in terms of initial attachment and subsequent proliferation (Wang et al., 2012b). Conflictingly, rat BMSCs were seeded on nano-fibers of PLA; while ALP activity and osteogenic gene expression (OP and OC) were similar, deposited calcium was significantly higher on aligned substrates at day 21 (Ma et al., 2005). Wang et al. (2012a)cultured rat MSCs on PHBHHx fibers, and saw markers of osteogenesis (osteocalcin and RUNX2) were higher on aligned fibers than random fibers in non-osteogenic media but in osteogenic media markers were expressed similarly. Martins et al. (2011) seeded hBMSCs on random and aligned electrospun PCL nanofibrous meshes and did not observe any differences in the expression of osteogenic genes but mineralized ECM was deposited along the fiber direction.

With regard to mature bone cells, mouse osteosarcoma cells were seeded on PLLA with nano- or micro-hydroxyapatite (HA) particles incorporated (Peng et al., 2011). Improved cell viability and superior ALP activity were observed on fibers with micro-HA over no-HA or nano-HA. However, aligned fibers with nanoHA had higher ALP than random fibers with nano-HA. These results could be due to a combination of surface roughness influence and biomimetic effect. In another study (Wang et al., 2011), decreased ALP activity and production of collagen type I and osteocalcin were observed with the increasing alignment of PLLA nanofibers when seeded with osteoblastic MG63 cells. Woo et al. (2003) observed enhanced differentiation of osteoblastic cells when seeded on scaffolds with rough struts compared with smooth struts and suggested this was due to increased cell adhesive protein (fibronectin and vitronectin) adsorption on rough surfaces. There are a number of reasons that could explain these differences between studies including the species of origin and the stage of maturity of the progenitor cell. Other scaffold properties may affect the outcome including pore size and fiber diameter, as well as substrate compliance, the material used, and the surface properties (chemistry and roughness).

Collagen production by hES-MPs was higher in random constructs with both media types compared with aligned fibers. SHG images of collagen for both media conditions also revealed a more intense signal from random fibrous constructs compared with aligned fibrous constructs. Although SHG signal intensity is influenced by several factors including fiber diameter, orientation, molecular organization, and collagen levels (Matcher, 2009), the lower SHG signal suggests reduced levels of collagen formation on these scaffolds. Interestingly there was an observable shift in the orientation of the matrix away from the direction of scaffold fiber orientation on aligned scaffolds from day 14 but the tensile modulus of the cell-seeded scaffolds continued to increase up to day 21. It has been shown previously that when the fiber deviation is <30° from the direction of externally applied tensile strain, this will not significantly affect the tensile modulus compared with no deviation (Nerurkar et al., 2009, 2011). This shift in orientation has also been observed in our laboratory using a different scaffold chemistry (polyurethane) whereby multiple layers of ECM deposited by MLO-A5s were aligned in a plane but differed in alignment between planes, producing a microstructure that resembles bone lamellae (Tetteh and Reilly, 2016). Such *in vitro* models of bone ECM can help us to better understand what controls collagen orientation in the bone matrix and hence how to manipulate this to produce stronger, more functional bone.

It is currently unclear how collagen is reorganized to create complex lamellae alignments in tissues but this seems to involve a combination of cell biochemical self-assembly and cell-mediated organization of the fibrils. An organelle that has recently been implicated in a variety of cell organizational and mechanotransduction mechanisms is the primary cilium (Satir et al., 2010). It has been observed *ex vivo* that primary cilia interact with ECM proteins in cartilage *via* integrins (Jensen et al., 2004), and organization has been shown to reflect cell polarity. In articular chondrocytes, cilia orientate away from the articular surface in cartilage and adopt orientations parallel to collagen fibrils (Donnelly et al., 2009) indicating that they may sense their surrounding environment and feedback to the cell nucleus *via* biological signals. In this study, we have preliminary evidence that primary cilia in bone progenitor cells orientate with the direction of scaffold fiber alignment as early as 3 days in culture, and this preceded collagen fiber deposition and alignment. However, this was not the case with the mature bone cells, which had a preference to orientate away from the principal axis of scaffold alignment and up to 20% were perpendicular. Our observation may be due to the maturity of the cell and the faster matrix deposition by MLO-A5s compared with hES-MPs, and therefore at the time of imaging, the cilia in MLO-A5s may no longer be required for matrix alignment but shift more to a sensory ECM maintenance phase, or even extending in search of an external mechanical stimulus such as fluid flow. Interestingly, primary cilia orientation found in rat tibial cortical osteocytes was shown to be mainly orientated perpendicular to the long axis of this weight-bearing bone (Uzbekov et al., 2012). To our knowledge, it has not yet been demonstrated whether cilia alignment has any primary influence on subsequent ECM deposition and alignment but this would be a very interesting area of future work, especially since the use of drugs that modify the primary cilia have recently been suggested as osteoporosis treatments.

Finally, we must acknowledge that there are some inherent limitations to this study. Electrospun scaffolds are essentially layers of densely packed fibers orientated in one plane resulting in diminishing pore size deeper into the scaffold layers. While the fiber diameter was the same for both scaffolds in this study, the size and shape of pores in random and aligned scaffolds were different. Pores within highly orientated fiber scaffolds tend to be longer and narrower compared with randomly orientated with those in randomly orientated mats. This is due to a higher fiber packing density and the way the fibers align. This is an uncontrolled variable in the study; however, a high packing density for the aligned fibers is necessary to offer structural integrity to the scaffold. It has, however, been demonstrated that the porosity of PCL fiber scaffolds with controlled geometry does directly affect hMSC osteogenic differentiation (Brennan et al., 2019). While our aligned configuration mimics the lamellae layers in bone, it would be useful in the future to use bioreactors to improve nutrient flow/waste removal. Secondly, a limitation of using hES-MPs and MLO-A5s as representative immature and mature bone cells, respectively, is that these cell lines come from different species. In addition, hES-MP cells are embryonic-derived and thus highly proliferative, but not immortal, while the MLO-A5 cell line has been immortalized. Therefore, the interesting differences we observed could be further verified using cells from the same donor or cell line at immature and mature stages of differentiation to elucidate mechanisms for the effects we have described.

## Conclusion

In conclusion, this study has shown that biomimetic scaffold fiber orientation can influence the osteogenesis of progenitor cells and the organization of deposited matrix. For the immature MSCs used in this study, differentiation was better supported by non-aligned fiber scaffolds; however as expected, the overall construct tensile strength in the fiber direction was higher for aligned fiber constructs. Our results indicate a complex relationship between external directional cues and osteogenic differentiation. It may be that the most suitable scaffold for osteogenesis of MSCs is a scaffold combining both random and orientated elements to enhance differentiation and control ECM organization.

## Data Availability Statement

The raw data supporting the conclusions of this article will be made available by the authors, without undue reservation.

## Author Contributions

RD-S undertook all experiments to analyze the data and wrote the first draft of the manuscript. AH undertook the literature review and wrote, edit, and format parts of the manuscript. NG supported the design and analysis the imaging sections of the work involving confocal and SHG imaging and edited the manuscript. GR was involved in all parts of the experimental design and analysis and undertook the manuscript writing and editing. All authors contributed to the article and approved the submitted version.

## Funding

This work was funded by the Engineering and Physical Sciences Research Council (EPSRC, EP/R513313/1; http://www.epsrc.ac.uk) on a doctoral training account to RD-S and AH from the department of Materials Science and Engineering, University of Sheffield. The funders had no role in the study design, data collection and analysis, and decision to publish, or preparation of the manuscript. The funders provide costs of open access publication *via* a block grant to the University of Sheffield.

## Conflict of Interest

The authors declare that the research was conducted in the absence of any commercial or financial relationships that could be construed as a potential conflict of interest.

## Publisher’s Note

All claims expressed in this article are solely those of the authors and do not necessarily represent those of their affiliated organizations, or those of the publisher, the editors and the reviewers. Any product that may be evaluated in this article, or claim that may be made by its manufacturer, is not guaranteed or endorsed by the publisher.
