# Supplementary figures and images for: Electrospun Fiber Alignment Guides Osteogenesis and Matrix Organization Differentially in Two Different Osteogenic Cell Types
Source: Front Bioeng Biotechnol. 2021 Oct 25;9:672959. doi: 10.3389/fbioe.2021.672959 (PMC8573409; doi:10.3389/fbioe.2021.672959)

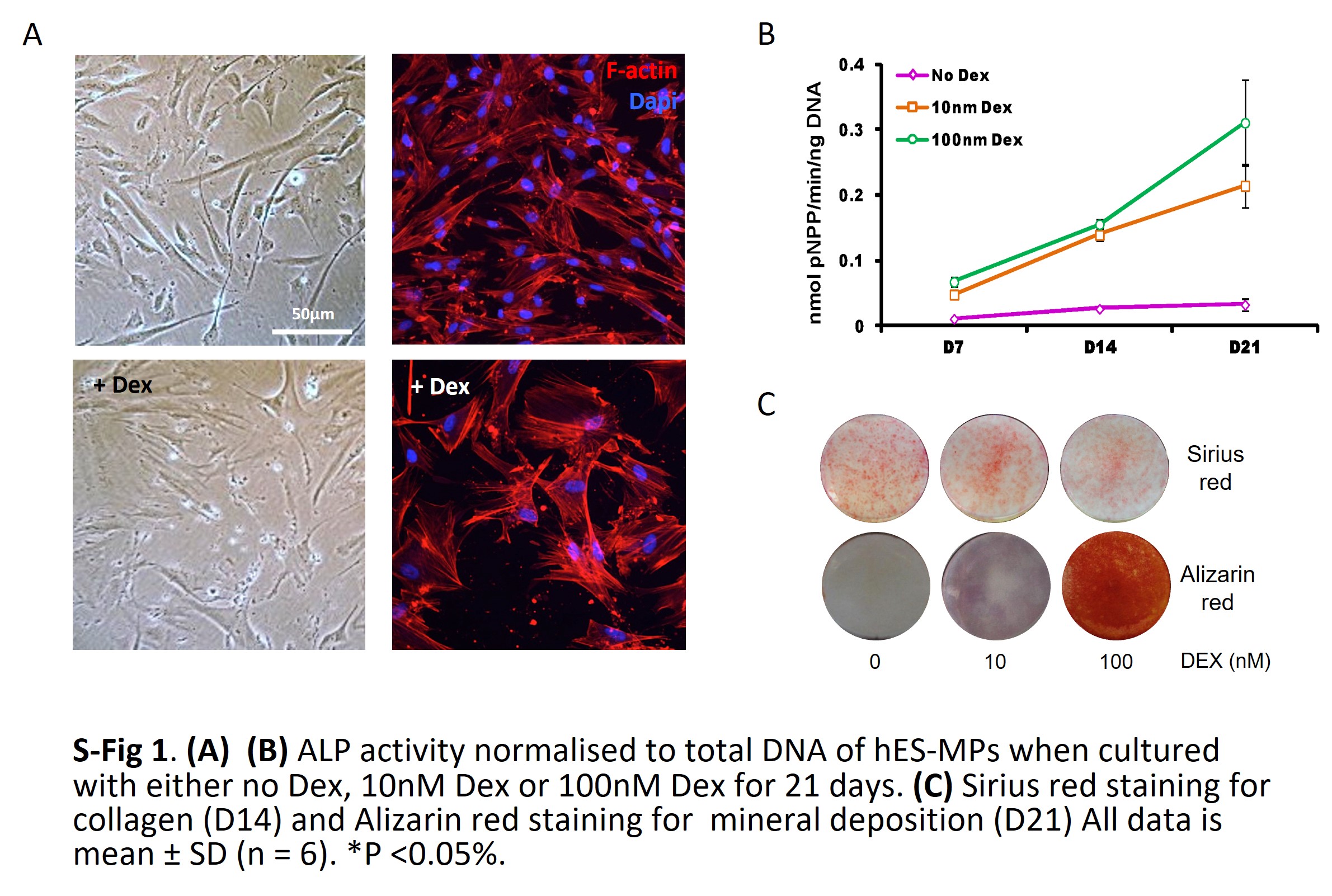

Supplement: Supplementary file 1 [file Image_1.jpg]

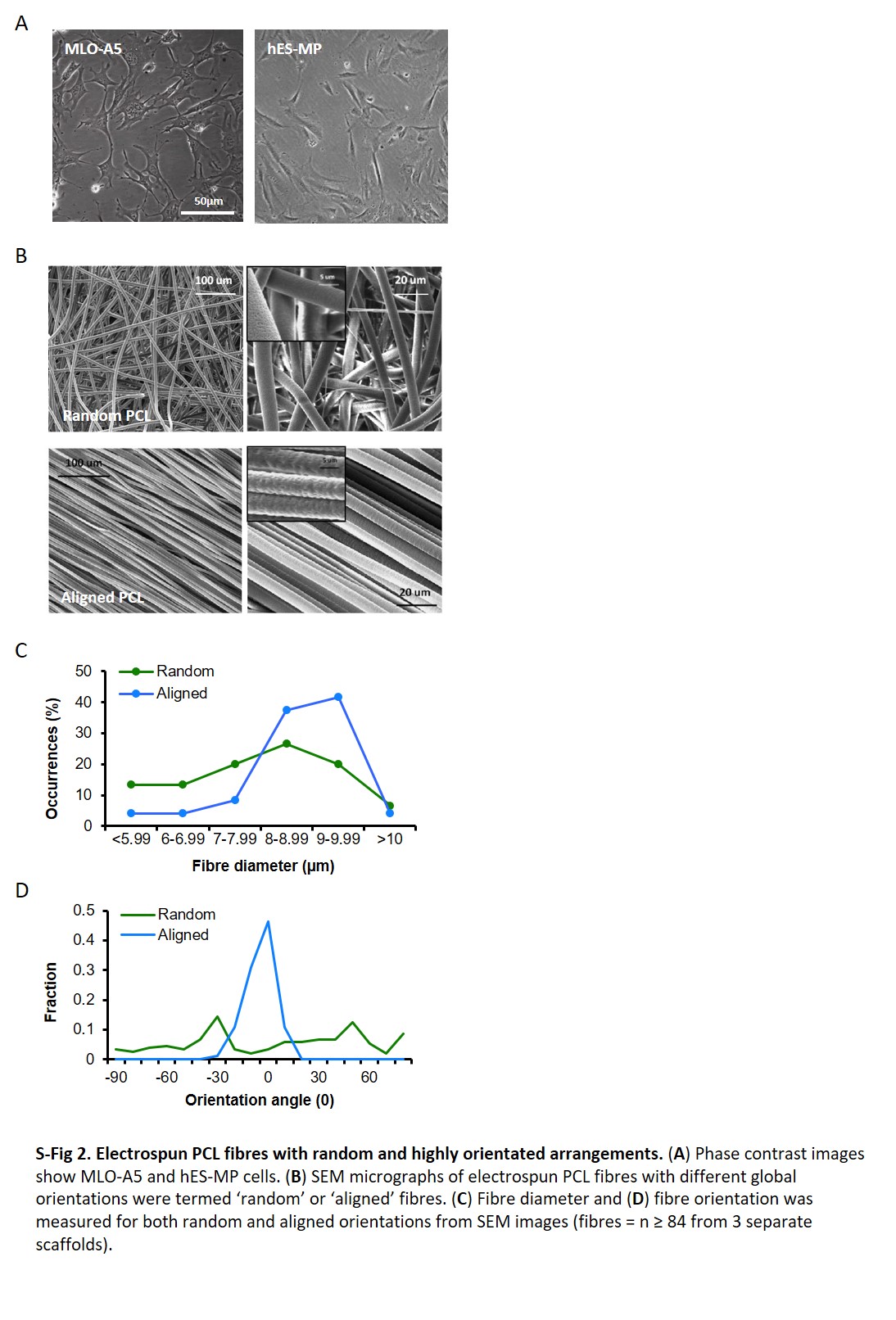

Supplement: Supplementary file 2 [file Image_2.jpg]

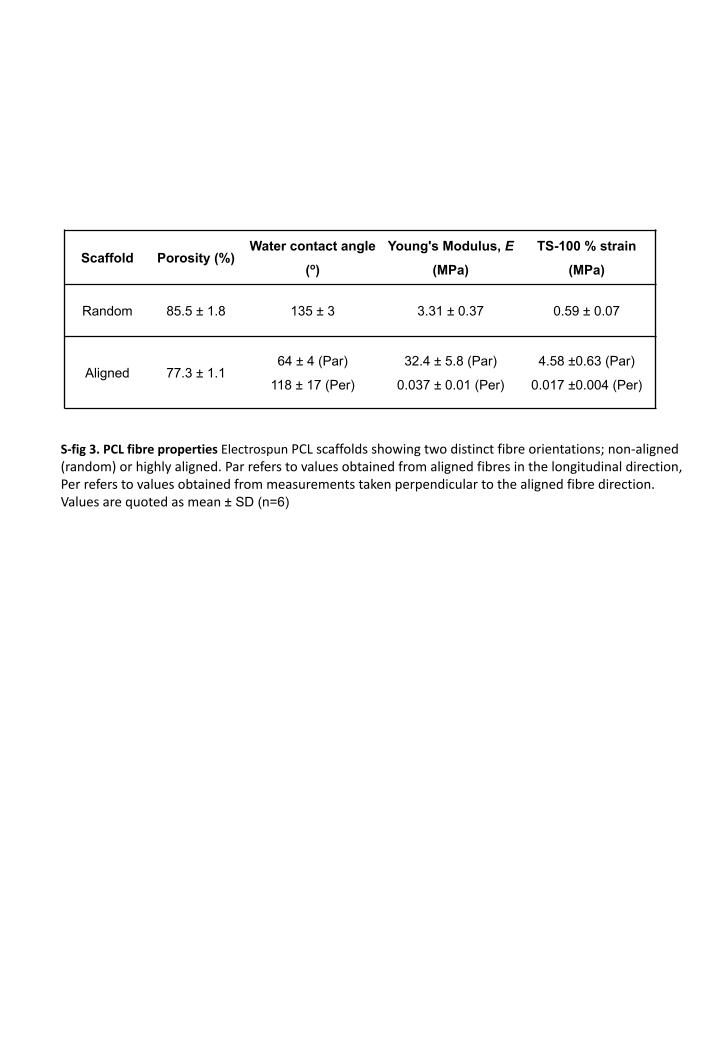

Supplement: Supplementary file 3 [file Image_3.JPEG]

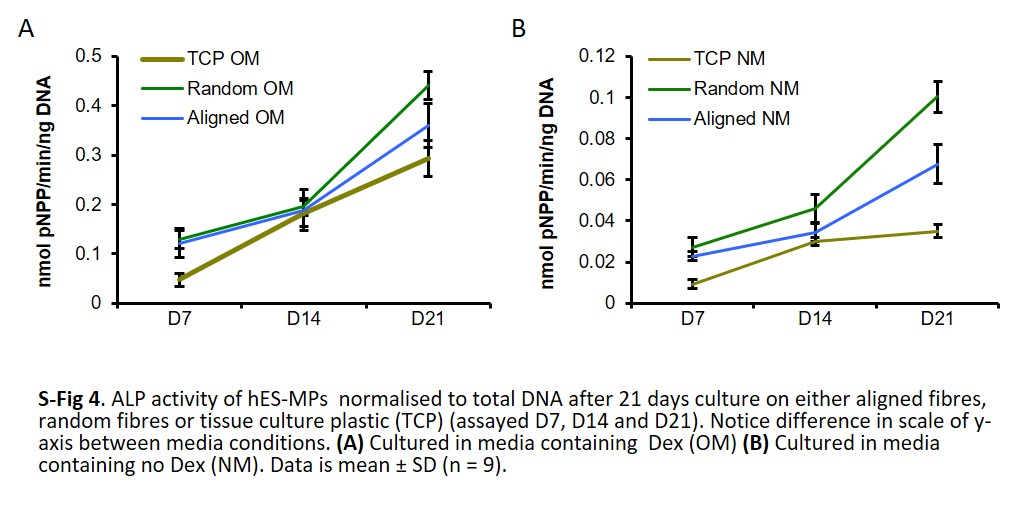

Supplement: Supplementary file 4 [file Image_4.JPEG]

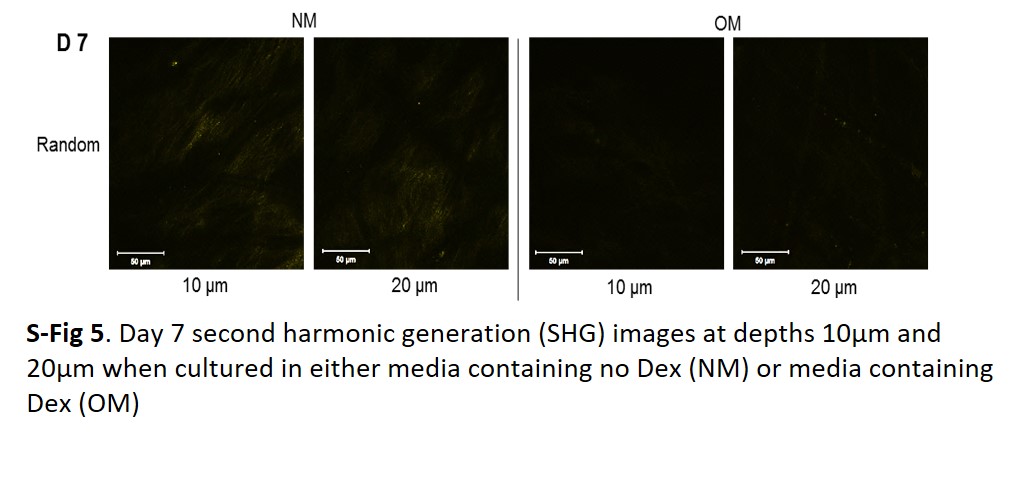

Supplement: Supplementary file 5 [file Image_5.jpg]
